# Dielectric and physicochemical characterization of Creole chicken breast meat for quality classification in the radiofrequency range

**DOI:** 10.1371/journal.pone.0349377

**Published:** 2026-05-19

**Authors:** Wilson Castro, Jorge Chavez, Lusmenia Ruiz-Mejía, Thony Arce, Himer Avila-George, Tony Chuquizuta

**Affiliations:** 1 Facultad de Ingeniería de Industrias Alimentarias y Biotecnología, Universidad Nacional de Frontera, Sullana, Piura, Perú; 2 Grupo de Investigación en Tecnologias Emergentes Fotónicas - GITEF, Instituto de Investigación del Mejoramiento Productivo, Universidad Nacional Autónoma de Chota, Chota, Cajamarca, Perú; 3 Centro de Investigación en Procesamiento Digital de Señales, Universidad de Guadalajara‌‌, Ameca, Jalisco, México; Yantai Institute of Technology, CHINA

## Abstract

Understanding the quality attributes of poultry meat is essential for food safety, consumer satisfaction, and value chain efficiency. This study presents a proof-of-concept assessment of the physicochemical and dielectric properties of Creole chicken breast meat to classify samples into three quality categories: PSE (pale, soft, exudative), RFN (reddish, firm, non-exudative), and DFD (dark, firm, dry). A total of 96 samples were classified using pH at 24 h postmortem, yielding 3.13% PSE, 54.17% RFN, and 42.70% DFD. Dielectric spectroscopy in the 20 Hz to 1 MHz range revealed group-dependent spectral differences, most notably between 40 Hz and 2 kHz, where DFD meat showed higher $\epsilon^{\prime }$ and $\epsilon^{\prime \prime }$ values under the evaluated conditions. The dielectric response was modeled using a two-dispersion approach, identifying α and β relaxation processes and differences in the α-relaxation frequency across categories (0.148 kHz for DFD, 0.105 kHz for RFN, and 0.127 kHz for PSE). Given the single-source sampling and the strong class imbalance, particularly the limited number of PSE samples, the findings should be interpreted as preliminary. Nevertheless, the results suggest that low-frequency dielectric parameters may be explored as candidates for rapid, non-destructive screening of meat quality categories, and they provide a reproducible workflow that can guide future validation studies and sensor-oriented designs in small-scale poultry production systems.

## Introduction

Poultry production has grown considerably in recent years worldwide. In Peru, poultry meat consumption represents 22.3% of the gross value of agricultural production, with chicken meat being the most representative (79%), positioning it as the largest source of animal protein nationwide [1]. The high demand for chicken meat is mainly due to its low cost [2] and its high protein content with high digestibility [3]. In addition, it contains low fat and beneficial polyunsaturated fatty acids, which contribute to the reduction of blood cholesterol [4].

Chicken meat is considered of good quality when the pH value is between 5.8 and 6.0 at 12 hours postmortem [5,6]. pH is a critical indicator of meat quality, together with other attributes such as flavor, color, freshness, and tenderness, which are highly valued by consumers [7]. Traditionally, internal quality attributes of chicken meat have been evaluated using physicochemical techniques to measure pH, color, water holding capacity (WHC), drip loss, cooking yield, and texture [8]. Although these techniques are accurate and reproducible, they are often invasive, require extensive sample processing, involve long procedures, and depend on chemical reagents and trained personnel [9]. These limitations hinder their application in routine quality control, especially in small-scale or rural poultry processing environments.

**Fig 1:**
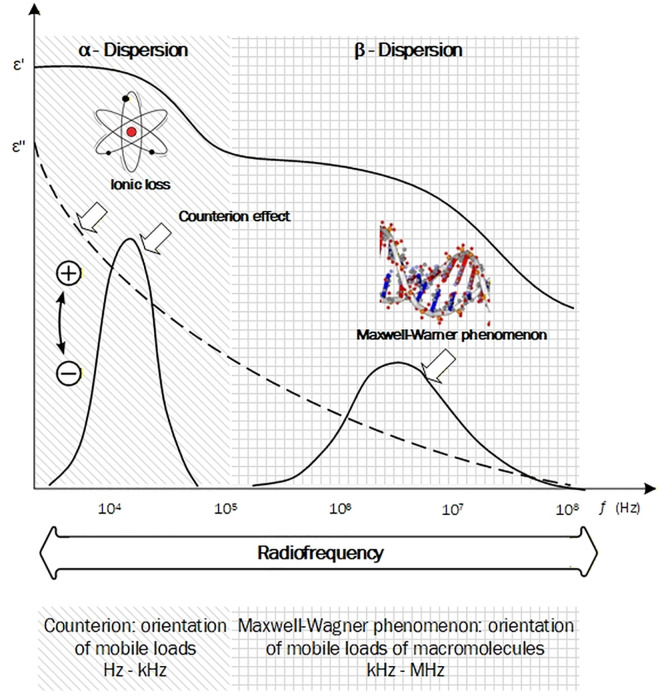
Physical and biological phenomena associated with the α and β dispersions in the radiofrequency range.

In response, the poultry industry is increasingly adopting modern, rapid, and non-destructive technologies for quality control. Among these, dielectric spectroscopy stands out as a promising technique due to its sensitivity to the water and ionic content of meat, and its capacity to provide real-time measurements [10,11]. According to [7,12], the permittivity is a vectorial property consisting of a real part ($\epsilon^{\prime }$), which is associated with the material’s capacity to store electrical energy, and an imaginary part ($\epsilon^{\prime \prime }$), which is related to dielectric losses. Within the radiofrequency range, the dielectric behavior facilitates the identification of two effects associated with molecular orientation (α and β dispersions) (see Fig 1). The α-dispersions (Hz–kHz range) have been shown to be associated with charged molecules that exhibit low mobility and high ionic strength, such as electrolytes and organic acids. In contrast, β-dispersions (kHz–MHz range) have been observed to be associated with charged macromolecules, such as proteins and carbohydrates, in addition to interactions on interfacial surfaces that exhibit charges with high surface tension [13]. These parameters vary with frequency, temperature, tissue density, and chemical composition [14,15].

Several authors have applied dielectric spectroscopy to study meat quality, including the characterization of the effects of aging in chicken [12], the detection of deep pectoral myopathy [9], and the classification of meats with different postmortem conditions [5,16]. However, most of these studies have focused on industrial breeds and commercial processing lines, using high-frequency ranges or microwave systems.

Despite this progress, there is limited research on the dielectric behavior of native or Creole chicken meat, which is widely produced in rural and small-scale systems across Latin America. Furthermore, few studies have jointly considered physicochemical attributes and radiofrequency dielectric modeling to explicitly describe α and β dispersion behavior in Creole chicken meat. This combination is relevant because α- and β-dispersions, associated with ionic polarization and interfacial/macromolecular effects, can capture structural and compositional differences linked to postmortem quality.

Therefore, this study presents an initial dielectric modeling of Creole chicken breast meat within the 40 Hz–1 MHz range, linking dielectric parameters with physicochemical attributes (pH, color, and moisture) to distinguish among meat quality categories. Although dielectric spectroscopy has previously been explored for poultry quality assessment, the evidence remains limited for Creole production systems and for low-frequency approaches that explicitly separate α and β dispersions within the radiofrequency range. Accordingly, the contribution of this work is incremental and primarily methodological: we couple a standard physicochemical classification (pH-based) with an interpretable two-dispersion dielectric modeling framework in a Creole chicken cohort, reporting relaxation-related parameters that can serve as baseline descriptors for future validation studies and sensor-oriented designs under broader commercial variability.

## Materials and methods

**Fig 2:**
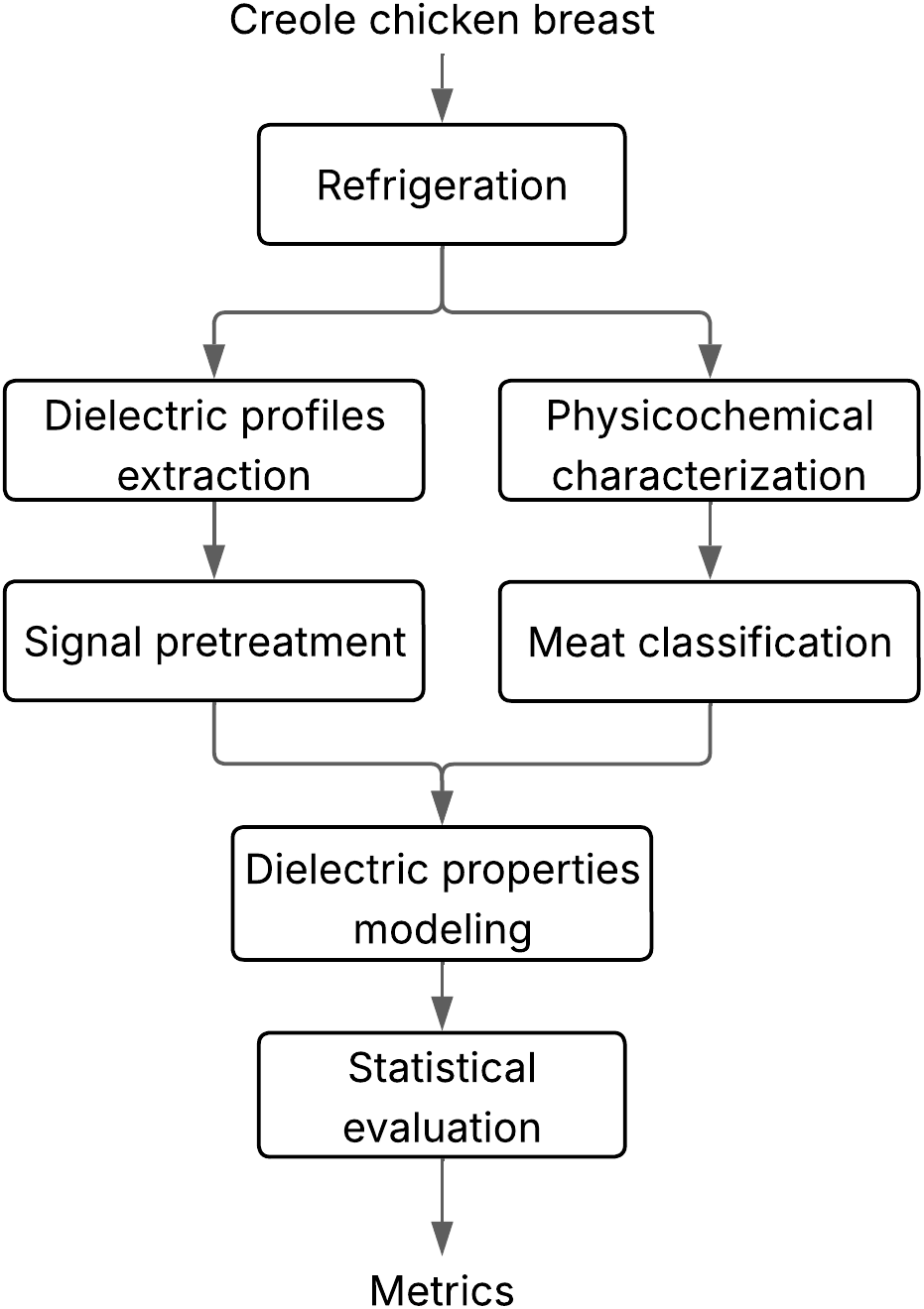
Experimental procedure for the physicochemical and dielectric characterization of chicken meat.

The experimental procedure is shown in Fig 2. Each step of the flowchart is explained in detail in the following paragraphs.

### Ethics statement‌‌

No ethical approval was required for this study. All meat samples were obtained from animals slaughtered under standard commercial procedures in a licensed processing facility. Live animal experimentation was not involved, and only postmortem tissues were analyzed.

### Samples

Ninety-six boneless and skinless male chicken breasts (Pectoralis major), aged 42–45 days, were obtained from the processing facility of the Multiservice Company DIR EIRL, located in Chota, Cajamarca, Peru. After slaughter, the birds were processed by bleeding, plucking, eviscerating, and chilling at 4 °C for 6 hours. The breast samples were then transported to the Laboratory for Emerging Technologies at the National Autonomous University of Chota in insulated containers (30×30×40 cm) containing frozen gel packets to maintain temperatures between 2 and 4 °C. Upon arrival, the samples were stored at 4 °C until reaching 8 h postmortem. Four samples were analyzed per day throughout the experiment.

### Dielectric profile acquisition

Dielectric measurements were performed using a parallel plate sensor (Keysight 16451B, Germany) connected to an impedance analyzer (Keysight E4990A). The equipment was powered on for 30 minutes before use to stabilize the electronic circuits. Calibration was performed in open-air and short-circuit configurations, following manufacturer recommendations to reduce measurement noise.

**Fig 3:**
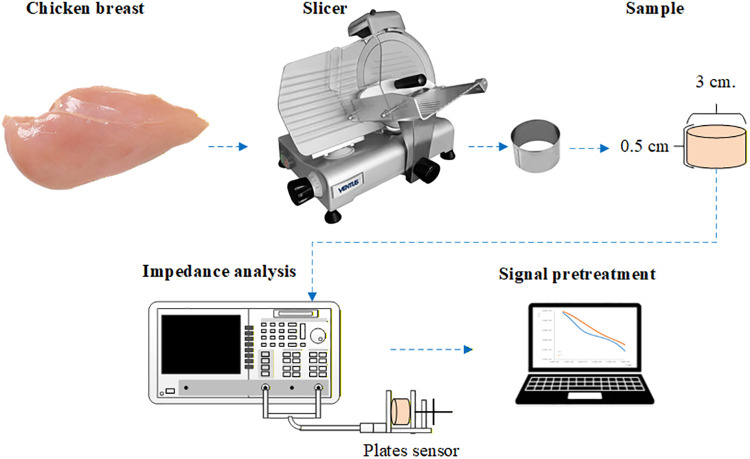
Extraction of dielectric properties of chicken meat in the radiofrequency range.

The samples were shaped into cylindrical forms using a laminator (Misato B250B3) and stainless-steel punch to ensure uniform contact with the electrodes (see Fig 3). The dielectric properties were measured from 40 Hz to 1 MHz using 401 discrete frequency points and a bandwidth of 1 kHz [17]. Five measurements were taken per sample at 8 hours postmortem.

### Signal pre-processing

From the measured parameters—the dissipation factor (*D*_*t*_) and the equivalent parallel capacitance (*C*_*p*_)—the dielectric constant ($\epsilon^{\prime }$) and the loss factor ($\epsilon^{\prime \prime }$) were calculated using Eqs. 1–3:


$$\begin{array}{c} \epsilon^{\prime }=\frac{t_{a}\cdot C_{p}}{\pi \cdot {\left(\frac{d}{a}\right)}^{2}\cdot \epsilon_{0}} \end{array}$$ (1)

$$\begin{array}{c} D_{t}=\tan\delta \end{array}$$ (2)

$$\begin{array}{c} \varepsilon^{\prime \prime }=\varepsilon^{\prime }\cdot D_{t} \end{array}$$ (3)

where *t*_*a*_ is the average thickness of the sample (m), *d* is the diameter of the upper electrode (38 mm), $\epsilon_{0}=8.854\times 10^{-12}$ F/m and δ is the phase angle of the dielectric response, representing the energy lost as heat.

### Physicochemical characterization

The samples were evaluated 8 and 24 hours after the test; pH was measured following AOAC method 981.12. For this, 5 g of sample were homogenized with 45 mL of distilled, filtered, and measured with a pH-meter (Orion VersaStar Pro, Thermo Scientific). Similarly, titratable acidity was determined according to the AOAC method 900.02 and expressed as lactic acid percentage using the Eq. 4.


$$\begin{array}{c} \text{Lactic acid }(\%)=100\cdot \frac{V_{\text{NaOH}}\cdot N_{\text{NaOH}}\cdot \text{Meq}}{\text{W}} \end{array}$$ (4)

where *V*_*NaOH*_ is the volumen of NaOH, *N*_*NaOH*_ is normal NaOH, *M*_*eq*_ is equivalent to Mili and W is the weight of the sample.

Finally, color was measured with a colorimeter (3Nh NR200) and moisture was determined by drying the samples at 110 °C for 48 hours (ISO 1442, 1997) to a constant weight.

### Meat quality classification

The meat was classified into three categories according to the pH at 24 hours postmortem following the standards proposed in previous studies [5,12], see Table 1

**Table 1:** Chicken meat quality classification parameter.

Meat type	pH
PSE (Pale, Soft, Exudative)	< 5.70
RFN (Red, Firm, Normal)	5.70–6.10
DFD (Dark, Firm, Dry)	≥ 6.10

### Modeling of dielectric properties

The average $\epsilon^{\prime }$ spectra for the three meat categories were fitted using a modified version of the model proposed by Traffano Schiffo et al. [14,18], as expressed in Eq. 5. This approach considers two dielectric dispersions (α and β) and was applied to a total of 59 spectra.

We adopted the two-dispersion equation derived from Traffano-Schiffo et al. because (i) it provides an interpretable separation of the dominant low-frequency (α) and higher-frequency (β) relaxation mechanisms (RF) commonly reported in muscle foods, (ii) it is parsimonious (few parameters) and thus less prone to overfitting than more flexible alternatives (e.g., multi-term Cole–Cole or Havriliak–Negami fits) for the present sample size and frequency grid, and (iii) it yields stable fits across spectra with excellent goodness-of-fit indicators. The model assumes that, within 40 Hz–1 MHz under the measurement conditions used, the dielectric response of $\epsilon^{\prime }$ can be reasonably approximated by two major dispersions and that residual errors around the fitted $\log(\epsilon^{\prime })$ curve are approximately independent. Parameters were estimated by nonlinear least squares; fit quality was quantified using *R*^2^ and RMSE (Eqs. 9–10), and the resulting relaxation frequencies are interpreted as effective (apparent) relaxation descriptors for the cohort. Alternative models were considered conceptually; however, the goal here is interpretability and parameter stability rather than maximizing flexibility.


$$\begin{array}{c} \log(\epsilon_{\left(\omega \right)}^{\prime })=\log(\epsilon_{\infty }^{\prime })+\sum_{n=1}^{2}\frac{\Delta \log(\epsilon_{n}^{\prime })}{1+e^{[\log(\omega^{2})-\log(\tau_{n}^{2})]\cdot \alpha_{n}}}. \end{array}$$ (5)

From this model, the dielectric constants associated with each dispersion and the corresponding relaxation frequencies were calculated using Eqs. 6–8:


$$\begin{array}{c} \epsilon_{\alpha }^{\prime }=10^{\log(\epsilon_{\infty }^{\prime })+\Delta \log(\epsilon_{\beta }^{\prime })+\frac{\Delta \log(\epsilon_{\alpha }^{\prime })}{2}} \end{array}$$ (6)

$$\begin{array}{c} \epsilon_{\beta }^{\prime }=10^{\log(\epsilon_{\infty }^{\prime })+\frac{\Delta \log(\epsilon_{\beta }^{\prime })}{2}} \end{array}$$ (7)

$$\begin{array}{c} f_{i}=10^{\frac{\log(\omega )\cdot \tau_{i}}{2}} \end{array}$$ (8)

where ω=2πf is the angular frequency, *f* is the linear frequency in Hz, and *i* corresponds to each dispersion (α and β).

To refine the model fitting in the radiofrequency region, the predicted values associated with the γ dispersion in the microwave range, obtained from the Traffano Schiffo equation, were incorporated into the analysis.

### Statistical analysis

Physicochemical data were analyzed using one-way analysis of variance followed by Tukey’s post hoc test at α=0.05, using Statgraphics Centurion XIX (Statgraphics Technologies Inc., USA). Spectral fitting was performed in the MATLAB 2023b software (MathWorks Inc.). To quantify the performance of each model, two complementary indicators were computed: the coefficient of determination (*R*^2^), presented in Eq. 9, and the root mean square error (RMSE), presented in Eq. 10. These expressions allow assessing, respectively, the proportion of variance explained by the model and the magnitude of the prediction error.


$$\begin{array}{c} R^{2}=1-\frac{\sum_{i=1}^{n}(y_{i}-\hat{y_{i}}{)}^{2}}{\sum_{i=1}^{n}(y_{i}-\bar{y}{)}^{2}} \end{array}$$ (9)

$$\begin{array}{c} RMSE=\sqrt{\frac{1}{n}\sum_{i=1}^{n}(\bar{y_{i}}-y_{i}{)}^{2}} \end{array}$$ (10)

where $\hat{y_{i}}$ and *y*_*i*_ are the predicted and actual values of $\varepsilon^{\prime }$ for the samples *i*^*th*^, and $\bar{y}$ is the average value of the actual value of $\varepsilon^{\prime }$ for the samples. The model that best fits is expected to have the highest *R*^2^ and the lowest value of *RMSE*.

### Inclusivity in global research

Additional information regarding the ethical, cultural, and scientific considerations specific to inclusivity in global research is included in the Supporting Information (S1 File).

## Results and discussion

### Physicochemical characterization of chicken meat

Table 2 summarizes the physicochemical characteristics of the chicken breast samples evaluated at 8 h and 24 h postmortem. Based on pH at 24 hours postmortem, the samples were classified into three categories: 3.13% PSE (n = 3), 54.17% RFN (n = 52) and 42.70% DFD (n = 41). Significant differences in pH were observed at 8 and 24 hours postmortem, as well as in humidity at 8 hours postmortem (*p* < 0.05). In contrast, no significant differences were found in the color (L*a*b*), acidity, or chroma values between the meat types.

**Table 2:** Physicochemical characterization of Creole chicken breast meat at 8 and 24 hours postmortem.

Parameter	Unit	PSE (n = 3)	RFN (n = 52)	DFD (n = 41)	p-value	CV (%)
pH 8 h pm	—	6.17 ± 0.02^*a*^	6.12 ± 0.14^*ab*^	6.21 ± 0.12^*b*^	0.074	2.24
Acidity 8 h pm	% lactic acid	1.05 ± 0.03^*a*^	1.16 ± 0.08^*a*^	1.96 ± 0.08^*a*^	0.939	7.47
Moisture 8 h pm	% (wet basis)	23.57 ± 0.50^*ab*^	23.90 ± 1.29^*b*^	23.05 ± 1.38^*a*^	0.010	5.83
L^*^ 8 h pm	—	55.54 ± 0.98^*a*^	53.44 ± 2.85^*a*^	53.14 ± 2.13^*a*^	0.281	4.77
a^*^ 8 h pm	—	3.20 ± 0.93^*a*^	3.70 ± 1.04^*a*^	3.90 ± 0.67^*a*^	0.305	23.89
b^*^ 8 h pm	—	10.25 ± 0.02^*a*^	9.69 ± 1.69^*a*^	10.24 ± 1.41^*a*^	0.252	16.21
*C* 8 h pm	—	11.30 ± 2.11^*a*^	10.66 ± 1.68^*a*^	11.22 ± 1.42^*a*^	0.229	14.58
*H* 8 h pm	deg	70.59 ± 0.02^*a*^	67.33 ± 3.46^*a*^	68.14 ± 3.55^*a*^	0.201	5.15
pH 24 h pm	—	5.37 ± 0.32^*a*^	5.96 ± 0.09^*b*^	6.21 ± 0.08^*c*^	0.000	3.28
L^*^ 24 h pm	—	53.90 ± 1.23^*a*^	53.89 ± 2.39^*a*^	53.39 ± 2.62^*a*^	0.620	4.58

Values are expressed as mean ± standard deviation. Different superscript letters within the same row indicate significant differences according to one-way ANOVA followed by Tukey’s post hoc test (α=0.05). PSE = pale, soft, exudative; RFN = reddish, firm, non-exudative; DFD = dark, firm, dry. CV = coefficient of variation.

**Fig 4:**
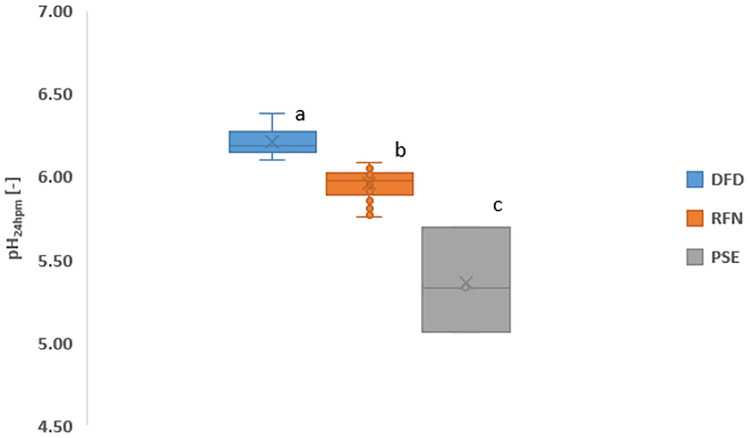
Classification of chicken breast meat based on pH values at 24 h postmortem.

Fig 4 shows the classification of meat samples based on the *pH*_24 *hours*_ postmortem. The PSE samples exhibited pH values below 5.70, which is consistent with the findings of Traffano-Schiffo et al. [9], Zhang and Barbut [16], and Li et al. [19]. The observed pH drop is associated with rapid anaerobic glycolysis and lactic acid production during postmortem metabolism [20,21], due to oxygen depletion and cellular anoxia [22]. This leads to increased protein denaturation, particularly sarcoplasmic and myofibrillar proteins [23,24].

**Fig 5:**
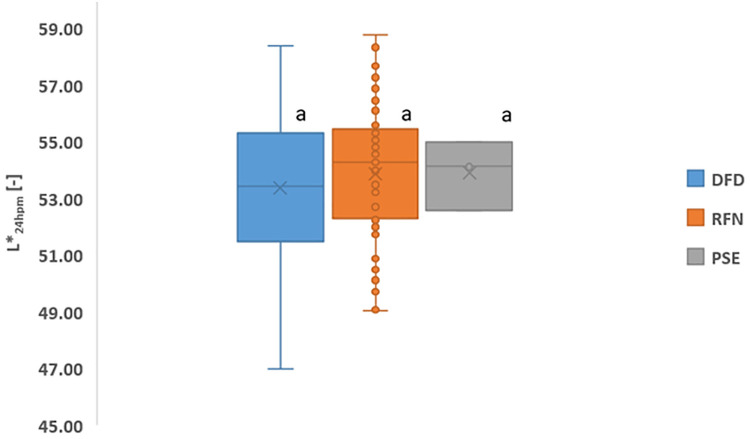
Lightness (L*) values of chicken meat types at 24 h postmortem.

Fig 5 illustrates the lightness (L*) values at 24 hours postmortem. No significant differences were found between the meat types, although slightly higher values were observed in the PSE samples. The variability in lightness can be attributed to differences in myoglobin concentration, water-holding capacity, and final pH [16,25]. Postmortem glycolysis influences muscle acidification and color variation [3,21,26].

### Classification of chicken meat

The frequency distribution of the meat quality types is summarized in Table 3. The meat of RFN was the most prevalent (54.17%), followed by DFD (42.70%) and PSE (3.13%). This distribution reflects the typical variability observed in small-scale poultry processing and highlights the usefulness of the *pH*_24 *hours*_ postmortem as a reliable quality indicator.

**Table 3:** Distribution of chicken meat types based on pH at 24 hours postmortem.

Type of meat	Observations (%)
PSE	3.0 (3.13%)
RFN	52.0 (54.17%)
DFD	41.0 (42.70%)

### Dielectric characterization of chicken meat

**Fig 6:**
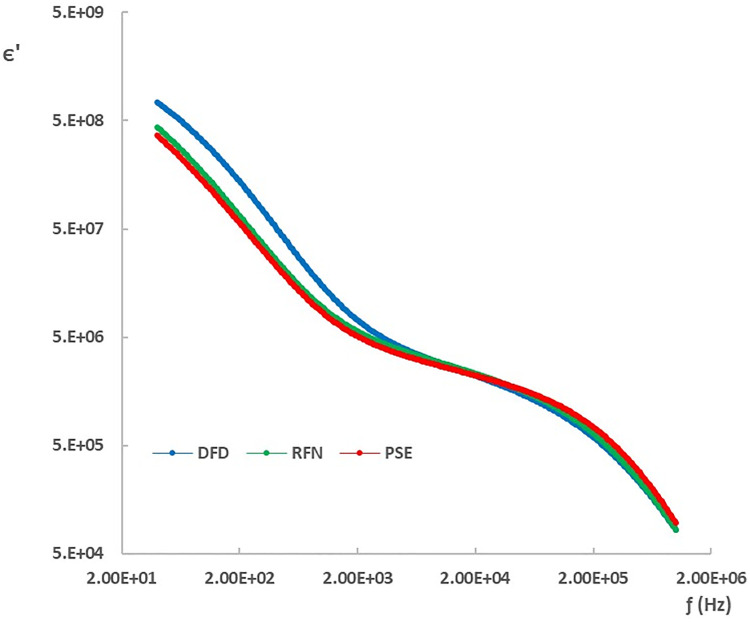
Dielectric constant spectra ($\epsilon^{\prime }$) of chicken meat types at 8 h postmortem.

**Fig 7:**
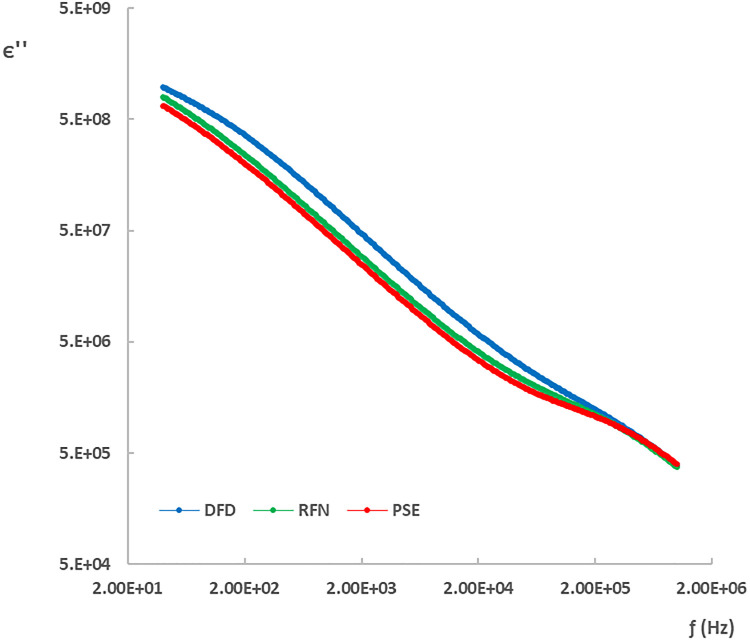
Dielectric loss factor spectra ($\epsilon^{\prime \prime }$) of chicken meat types at 8 h postmortem.

Figs 6 and 7 show the dielectric constant ($\epsilon^{\prime }$) and loss factor ($\epsilon^{\prime \prime }$) spectra for each type of meat in the frequency range of 20 Hz to 1 MHz at 8 hours postmortem. Both $\epsilon^{\prime }$ and $\epsilon^{\prime \prime }$ decrease with increasing frequency, as expected in biological tissues. In particular, the dielectric properties of DFD meat differed from those of PSE and RFN in the low-frequency region (40 Hz to 2 kHz, corresponding to α-dispersion). This behavior is consistent with postmortem differences in ionic strength, membrane polarization, and intra- and extracellular water distribution [5,10,27], as well as intracellular and extracellular water dynamics [9,12].

The observed separation in the α-dispersion region (approximately 40 Hz–2 kHz) is consistent with postmortem differences in ionic strength and water compartmentalization driven by glycolysis and pH decline [20,21]. In PSE-like trajectories, accelerated glycolysis and lactate accumulation increase the concentration of mobile ions and modify membrane-associated polarization, which can elevate the low-frequency dielectric response [5,22,27]. In contrast, DFD meat is associated with reduced glycogen availability and a higher ultimate pH, which can alter protein–water interactions and the balance between intra- and extracellular water, affecting interfacial polarization processes [7,12]. In the β-dispersion range, dielectric losses are more influenced by dipolar relaxation and macromolecular’s phenom (example: protein and carbohydrate degradation or transformations); protein denaturation and changes in water binding can modulate effective permittivity and relaxation behavior [9,23,24]. Therefore, dielectric parameters can be interpreted as indirect cohort-level markers of the biochemical and structural state of the muscle during early postmortem time [5,10].

At the cellular level, the dielectric properties are influenced by the polarization of the membrane and the organization of ions across the cell wall, which acts as a capacitor under alternating electric fields [9,12]. In the β-dispersion range, losses are dominated by the polarization of the water molecules and dipolar excitation [5,28]. Above 4 GHz, the dominant mechanism is water polarization, where PSE samples have been shown to exhibit higher values of $\epsilon^{\prime }$ than RFN [14].

### Modeling dielectric spectra of chicken meat

Table 4 presents the modeling parameters obtained from the dielectric spectra of the three types of meat. The PSE meat exhibited a higher β-dispersion dielectric constant (4.836 ± 0.129) and faster relaxation ($\tau_{\beta }=7.236\pm 0.024$), indicative of higher protein denaturation and ionic mobility [9].

**Table 4:** Fitted parameters of the dielectric spectra model for Creole chicken breast meat.

Parameter	Unit	Description	DFD	RFN	PSE
$\varepsilon_{\infty }^{\prime }$	—	Dielectric constant at infinite frequency	1.251 ± 0.289	1.158 ± 0.286	1.110 ± 0.018
$\Delta \log(\varepsilon_{\alpha }^{\prime })$	—	Magnitude of α-dispersion	2.986 ± 0.171	3.246 ± 0.750	3.042 ± 0.0144
$\tau_{\alpha }$	$\log({Hz}^{-1})$	Relaxation time for α-dispersion	3.107 ± 0.241	2.709 ± 0.602	2.905 ± 0.001
$\alpha_{\alpha }$	—	Broadening factor for α-dispersion	0.301 ± 0.019	0.299 ± 0.029	0.282 ± 0.001
$\Delta \log(\varepsilon_{\beta }^{\prime })$	—	Magnitude of β-dispersion	4.963 ± 0.157	4.947 ± 0.154	4.789 ± 0.136
$\tau_{\beta }$	$\log({Hz}^{-1})$	Relaxation time for β-dispersion	7.240 ± 0.089	7.220 ± 0.069	7.250 ± 0.005
$\alpha_{\beta }$	—	Broadening factor for β-dispersion	0.102 ± 0.009	0.108 ± 0.010	0.121 ± 0.001
*R* ^2^	—	Coefficient of determination of model fit	0.999 ± 0.00004	0.999 ± 0.00003	0.999 ± 0.000001
*RMSE*	—	Root mean square error	0.006 ± 0.005	0.005 ± 0.004	0.003 ± 0.0003

Values are expressed as mean ± standard deviation based on averaged spectra of each meat type (n = 41 for DFD, n = 52 for RFN, n = 3 for PSE). The two-dispersion model (α and β) was fitted by nonlinear least squares in MATLAB 2023b. Parameters α and β refer to low- and high-frequency dispersions, respectively.

**Fig 8:**
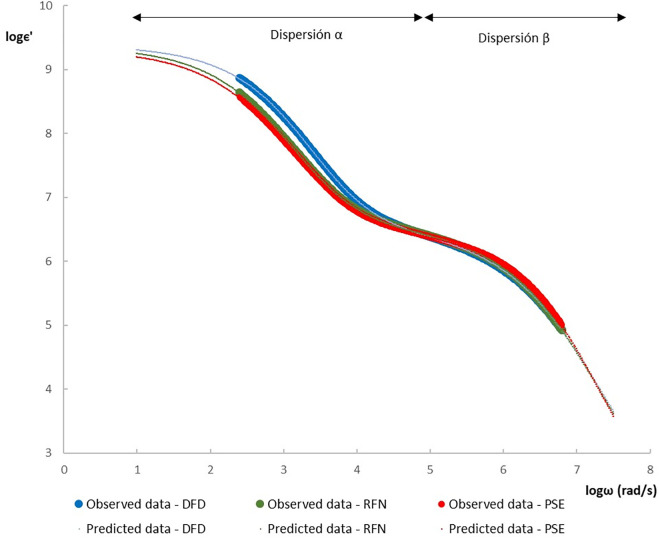
Modeling of dielectric spectra for each type of Creole chicken meat.

Fig 8 illustrates the modeled spectra, highlighting two relaxation processes: α and β. The parameters of the fitted model provide important information on the dielectric mechanisms underlying the behavior of the postmortem muscle. The α-dispersion, located in the low-frequency range (40 Hz–2 kHz), is mainly associated with the polarization of bound water and the movement of small ions such as lactate and electrolytes, reflecting the metabolic state of the tissue. In contrast, the β -dispersion (kHz–MHz) represents dipolar relaxation processes of macromolecules, including myofibrillar proteins and collagen, which are influenced by protein denaturation and water–protein interactions [9,10,14]. These findings indicate that dielectric relaxation parameters reveal molecular and structural differences among the PSE, RFN, and DFD meat types.

The dielectric constants and relaxation frequencies of both dispersions are summarized in Table 5. Although no statistically significant differences were observed, the PSE samples tended to exhibit higher $\epsilon^{\prime }$ values in the β-dispersion region, suggesting an increase in lactate and phosphate content. In contrast, DFD meat showed the lowest $\epsilon^{\prime }$ values, likely due to limited glycolytic activity and reduced ion production [5,7,9]. Relaxation frequencies ($f_{\alpha }$ and $f_{\beta }$) further reflect differences in ionic mobility and water distribution between meat types: DFD meat exhibited the slowest α-relaxation (0.148 kHz), while PSE samples exhibited faster relaxation associated with greater ion mobility and lactate accumulation. These dielectric patterns are consistent with postmortem biochemical processes and reinforce the potential of model parameters as discriminative indicators of meat quality [5,9,29].

**Table 5:** Dielectric constants ($\varepsilon^{\prime }$) and relaxation frequencies (*f*) of Creole chicken breast meat for α and β dispersions.

Parameter	Unit	Description	DFD	RFN	PSE
$\varepsilon_{\alpha }^{\prime }\times 10^{7}$	—	Dielectric constant of α-dispersion	6.46 ± 3.49^*a*^	7.63 ± 7.26^*a*^	2.64 ± 0.278^*a*^
$\varepsilon_{\beta }^{\prime }\times 10^{3}$	—	Dielectric constant of β-dispersion	6.56 ± 3.38^*a*^	5.31 ± 2.77^*a*^	3.21 ± 0.037^*a*^
$f_{\alpha }$	kHz	Relaxation frequency of α-dispersion	0.232 ± 0.150^*a*^	0.123 ± 0.009^*b*^	0.128 ± 0.003^*ab*^
$f_{\beta }$	MHz	Relaxation frequency of β-dispersion	2.821 ± 0.606^*a*^	2.678 ± 0.473^*a*^	2.832 ± 0.032^*a*^

Values are expressed as mean ± standard deviation based on averaged spectra per meat type (n = 3 for PSE, n = 52 for RFN, n = 41 for DFD).

Different superscript letters within each row indicate significant differences according to one-way ANOVA followed by Tukey’s test (α = 0.05).

α and β refer to the low- and high-frequency relaxation processes, respectively.

From an applied perspective, these results suggest that low-frequency dielectric parameters could be explored as candidates for rapid screening of meat quality categories under controlled measurement conditions. However, the present study is limited by single-facility sampling and strong class imbalance (particularly the small PSE subset) for using chemometrics models, which constrains statistical power and generalizability between breeds, processing lines, and commercial variability. Therefore, any practical deployment would require broader validation, including multi-breed and multi-site datasets, repeatability and reproducibility studies across operators and instruments, and external test sets collected under realistic handling and temperature variability (conservations temperature). In this context, the main value of the present work is providing a reproducible measurement and modeling workflow and reporting dispersion parameters that can guide future sensor-oriented validation efforts, rather than demonstrating immediate readiness for routine industrial implementation.

This study used samples from a single processing facility and a single breed/sex/age window, and dielectric measurements were performed under controlled laboratory handling. The class distribution was highly imbalanced (notably, a very small subset of PSE), which limits statistical power for that category and constrains generalization between breeds, processing lines, and commercial variability. Consequently, the findings should be interpreted as preliminary and hypothesis-generating; a more thorough validation with balanced classes, multiple breeds, multiple facilities, and external test sets is required before considering routine industrial use.

## Conclusions

The intensity values of the dielectric spectra ($\epsilon^{\prime }$ and $\epsilon^{\prime \prime }$) distinguished DFD meat from PSE and RFN meat in the low-frequency range of 40 Hz to 2 kHz at 24 h postmortem under the evaluated conditions. This differentiation is consistent with postmortem metabolic pathways that influence the physicochemical characteristics of Creole chicken meat. Among the 96 samples analyzed, three were classified as PSE, 52 as RFN, and 41 as DFD based on pH values at 24 h postmortem. Although significant differences in pH and moisture (%) were observed, particularly at 8 and 24 h, no statistical differentiation was found in color values (L*a*b*), acidity, or lightness between meat types. These results suggest that dielectric spectroscopy may be more sensitive to postmortem metabolic changes than the physicochemical parameters evaluated in this study.

The dielectric spectra ($\epsilon^{\prime }$) for each meat type were modeled using a two-dispersion approach, yielding distinct α-dispersion relaxation frequencies: 0.148 ± 0.003 kHz for DFD, 0.105 ± 0.002 kHz for RFN, and 0.127 ± 0.002 kHz for PSE. Overall, the results support the *potential* of low-frequency dielectric modeling as a rapid, non-destructive approach for screening meat quality categories in Creole chicken meat under controlled laboratory handling. However, given the single-facility sampling and the strong class imbalance (particularly the limited PSE subset) for using chemometrics models, the findings should be interpreted as preliminary.

Future work should validate robustness across breeds and facilities, assess repeatability across operators and instruments, and evaluate external predictive performance using independent test sets under commercial variability before translation to field-ready devices. Additional studies should also examine dielectric behavior beyond 24 hours postmortem to better capture postmortem biochemical transformations.

## Supporting information

S1 File Inclusivity in global research questionnaire. This file contains the completed Inclusivity in Global Research Questionnaire, which documents ethical, cultural, and scientific considerations relevant to studies that involve external communities, field activities, or cross-cultural contexts.
(DOCX)

## References

[1] Ministerio de Desarrollo Agrario y Riego. Production and marketing of poultry products. 2024. http://hdl.handle.net/20.500.13036/1683

[2] Petracci M, Mudalal S, Soglia F, Cavani C. Meat quality in fast-growing broiler chickens. World’s Poultry Science Journal. 2015;71(2):363–74. doi: 10.1017/s0043933915000367

[3] Zaboli G, Huang X, Feng X, Ahn DU. How can heat stress affect chicken meat quality? - a review. Poult Sci. 2019;98(3):1551–6. doi: 10.3382/ps/pey399 30169735

[4] Gallinger C, Federico F, Pighin D, Cazaux N, Trossero M, Marsó A. Determination of the nutritional composition of Argentinean chicken meat. Diaeta. 2016;34(156):10–8.

[5] Traffano‐Schiffo MV, Chuquizuta T, Castro‐Giraldez M, Fito PJ. Development of a methodology to categorize poultry meat affected by deep pectoral myopathy. J Food Process Preserv. 2021;45(3). doi: 10.1111/jfpp.15226

[6] León M, Orduz A, Velandia M. Physicochemical composition of sheep, chicken, beef, and pork meat. Ciencia y Tecnología Alimentaria. 2018;15(2):62–75. doi: 10.24054/16927125.v2.n2.2017.2969

[7] Zhuang H, Nelson SO, Trabelsi S, Savage EM. Dielectric properties of uncooked chicken breast muscles from ten to one thousand eight hundred megahertz. Poult Sci. 2007;86(11):2433–40. doi: 10.3382/ps.2006-00434 17954595

[8] Beshah W. Pork meat quality, effect of freezing and thawing, use of dielectric heating for thawing and dielectric spectroscopy to assess technological quality. Barcelona, Spain: Universitat Autónoma de Barcelona. 2014.

[9] Traffano-Schiffo MV, Castro-Giraldez M, Colom RJ, Fito PJ. Innovative photonic system in radiofrequency and microwave range to determine chicken meat quality. Journal of Food Engineering. 2018;239:1–7. doi: 10.1016/j.jfoodeng.2018.06.029

[10] Castro-Giráldez M, Botella P, Toldrá F, Fito P. Low-frequency dielectric spectrum to determine pork meat quality. Innovative Food Science & Emerging Technologies. 2010;11(2):376–86. doi: 10.1016/j.ifset.2010.01.011

[11] Sosa-Morales ME, Valerio-Junco L, López-Malo A, García HS. Dielectric properties of foods: Reported data in the 21st Century and their potential applications. LWT - Food Science and Technology. 2010;43(8):1169–79. doi: 10.1016/j.lwt.2010.03.017

[12] Trabelsi S, Roelvink J, Russell RB. Investigating the Influence of Aging on Radiofrequency Dielectric Properties of Chicken Meat. Journal of Microwave Power and Electromagnetic Energy. 2014;48(4):215–20. doi: 10.1080/08327823.2014.11689885

[13] Chuquizuta T, Castro W, Castro-Giraldez M, Fito PJ. Non-invasive monitoring of goldenberry freezing using infrared thermography and radiofrequency dielectric spectroscopy. Innovative Food Science & Emerging Technologies. 2025;104:104134. doi: 10.1016/j.ifset.2025.104134

[14] Traffano-Schiffo MV, Castro-Giraldez M, Colom RJ, Fito PJ. Development of a Spectrophotometric System to Detect White Striping Physiopathy in Whole Chicken Carcasses. Sensors (Basel). 2017;17(5):1024. doi: 10.3390/s17051024 28471378 PMC5469629

[15] Zainal Abidin Z, Omar FN, Biak DRA, Man YC. Alternative for Rapid Detection and Screening of Pork, Chicken, and Beef Using Dielectric Properties in the Frequency of 0.5 to 50 GHz. International Journal of Food Properties. 2015;19(5):1127–38. doi: 10.1080/10942912.2015.1058274

[16] Zhang L, Barbut S. Rheological characteristics of fresh and frozen PSE, normal and DFD chicken breast meat. Br Poult Sci. 2005;46(6):687–93. doi: 10.1080/00071660500391516 16428111

[17] Castro W, Gamboa P, Ruiz-Mejía L, Arce T, Avila-George H, Chuquizuta T. Dataset of dielectric spectra and physicochemical parameters of Creole chicken breast meat. Data Brief. 2025;63:112183. doi: 10.1016/j.dib.2025.112183 41210353 PMC12595077

[18] Traffano-Schiffo MV, Castro-Giraldez M, Colom RJ, Talens P, Fito PJ. New methodology to analyze the dielectric properties in radiofrequency and microwave ranges in chicken meat during postmortem time. Journal of Food Engineering. 2021;292:110350. doi: 10.1016/j.jfoodeng.2020.110350

[19] Li S, Xu X, Zhou G. The roles of the actin-myosin interaction and proteolysis in tenderization during the aging of chicken muscle. Poult Sci. 2012;91(1):150–60. doi: 10.3382/ps.2011-01484 22184440

[20] Damez J-L, Clerjon S, Abouelkaram S, Lepetit J. Beef meat electrical impedance spectroscopy and anisotropy sensing for non-invasive early assessment of meat ageing. Journal of Food Engineering. 2008;85(1):116–22. doi: 10.1016/j.jfoodeng.2007.07.026

[21] Huang M, Huang F, Xue M, Xu X, Zhou G. The effect of active caspase-3 on degradation of chicken myofibrillar proteins and structure of myofibrils. Food Chem. 2011;128(1):22–7. doi: 10.1016/j.foodchem.2011.02.062 25214324

[22] Adzitey F, Nurul H. Pale soft exudative (PSE) and dark firm dry (DFD) meats: causes and measures to reduce these incidences-a mini review. International food research journal. 2011;18(1).

[23] Gomez-Portilla M, Gomez N, Martínez-Benavides J. Evaluación de las características organolépticas, físicas y químicas de pechuga de pollo, en San Juan de Pasto (Nariño). Revista Veterinaria y Zootecnia. 2016;10(2):62–71. doi: 10.17151/vetzo.2016.10.2.6

[24] Huang M, Huang F, Ma H, Xu X, Zhou G. Preliminary study on the effect of caspase-6 and calpain inhibitors on postmortem proteolysis of myofibrillar proteins in chicken breast muscle. Meat Sci. 2012;90(3):536–42. doi: 10.1016/j.meatsci.2011.09.004 22098823

[25] Huff Lonergan E, Zhang W, Lonergan SM. Biochemistry of postmortem muscle - lessons on mechanisms of meat tenderization. Meat Sci. 2010;86(1):184–95. doi: 10.1016/j.meatsci.2010.05.004 20566247

[26] Hernández Bautista J, Aquino López JL, Ríos Rincón FG. Pre-mortem handling effect on the meat quality. Nacameh. 2013;7(2):41–64. doi: 10.24275/uam/izt/dcbs/nacameh/2013v7n2/hernandez

[27] Trabelsi S. Variation of the dielectric properties of chicken meat with frequency and temperature. Food Measure. 2015;9(3):299–304. doi: 10.1007/s11694-015-9235-6

[28] Bhat ZF, Morton JD, Mason SL, Bekhit AE-DA. Role of calpain system in meat tenderness: A review. Food Science and Human Wellness. 2018;7(3):196–204. doi: 10.1016/j.fshw.2018.08.002

[29] Traffano-Schiffo MV, Castro-Giraldez M, Herrero V, Colom RJ, Fito PJ. Development of a non-destructive detection system of Deep Pectoral Myopathy in poultry by dielectric spectroscopy. Journal of Food Engineering. 2018;237:137–45. doi: 10.1016/j.jfoodeng.2018.05.023

