# Supplementary material for: Dielectric and physicochemical characterization of Creole chicken breast meat for quality classification in the radiofrequency range
Source: PLoS One. 2026 May 19;21(5):e0349377. doi: 10.1371/journal.pone.0349377 (PMC13186348; doi:10.1371/journal.pone.0349377)
Supplement: S1 File — This file contains the completed Inclusivity in Global Research Questionnaire, which documents ethical, cultural, and scientific considerations relevant to studies that involve external communities, field activities, or cross-cultural contexts. (DOCX) [file pone.0349377.s001.docx]

Inclusivity in global research

PLOS’ policy on inclusivity in global research aims to improve transparency in the reporting of research performed outside of researchers’ own country or community and ensures that PLOS publications reporting global research adhere to high standards for research ethics and authorship. Authors of relevant research articles may be asked to complete the questionnaire below, which outlines ethical, cultural, and scientific considerations specific to inclusivity in global research. This questionnaire may be requested when researchers have travelled to a different country to conduct research, if research uses samples collected in another country, research with Indigenous populations or their lands, or if research is on cultural artefacts. Researchers travelling to another country solely to use laboratory equipment will not normally be required to complete the questionnaire. However, the questionnaire can be requested at the journal’s discretion for any submission – if you have been requested to complete this questionnaire by the PLOS journal you submitted to, please do so.

The questions have been designed to be applicable to a wide range of study types, and there are subsections for both human subjects research and non-human subjects research. If any of the questions are not relevant to your research please mark them as “N/A” as appropriate.

**Ethical considerations, permits and authorship**

*This section is applicable to all research types.*

Provide details as to who granted permissions and/or consent for the study to take place in the Methods section of your manuscript. This should include the names of **all** ethics boards, governmental organizations, community leaders or other bodies that provided approval for the study. If individuals provided approval refer to these people by their role or title but do not list their name(s).

Reported on page number: 3

Based on the procedures described in the manuscript, all activities were conducted exclusively on postmortem chicken breast samples obtained from the licensed commercial processing facility Multiservice Company DIR EIRL (Chota, Cajamarca, Peru). No live animals were handled, and no experimental interventions were performed on animals or humans. Because the study involved only commercially obtained postmortem tissues and did not include human participants or identifiable personal data, approval from an institutional ethics board, including the ethics committee of the Universidad Nacional Autónoma de Chota, was not required. Permission to access and analyze the samples was granted by the management of the processing facility in accordance with standard commercial procedures.

If there were any deviations from the study protocol after approval was obtained please provide details of these changes in the Methods section of your manuscript.

N/A

Did this study involve local collaborators that are residents of the country where the research was conducted or members of the community studied? If you do not have any authors from said communities, please provide an explanation for this below.

Yes. The study involved local collaborators who are residents of Peru, where the chicken meat samples were obtained and the physicochemical measurements were carried out. These collaborators contributed directly to sample acquisition, laboratory analyses, and interpretation of the results, and they are included as co-authors of the manuscript.

The study did not involve human communities, cultural groups, or Indigenous populations, because the work focused exclusively on postmortem chicken breast samples obtained from a commercial processing facility. As no community was the subject of the research, community-based authorship or representation was not applicable.

This study did not involve human communities, cultural groups, or Indigenous populations, as the research was conducted exclusively on postmortem chicken breast samples obtained from a commercial processing facility. Therefore, no community-based authorship or representation was applicable.

Everyone listed as an author should meet PLOS’ criteria for authorship and all individuals who meet these criteria should be included in the author byline, rather than the acknowledgements. For further information please see the journal’s Authorship Policy.

**Human subjects research (e.g. health research, medical research, cross-cultural psychology)**

Did you obtain written informed consent from a representative of the local community or region before the research took place? How did you establish who speaks for the community? Details of written informed consent obtained from study participants should be reported separately in the Methods section of your manuscript.

N/A

How did members of the local community provide input on the aims of the research investigation, its methodology, and its anticipated outcome(s)?

N/A

When engaging with the local community, how did you ensure that the informed consent documents and other materials could be understood by local stakeholders?

N/A

Will the findings of the research be made available in an understandable format to stakeholders in the community where the study was conducted (e.g. via a presentation, summary report, copies of publications, etc.)? Please provide details of how this will be achieved.

N/A

**Non-human subjects research using specimens/ animals collected as part of the study, or those housed in archival collections. Examples include archaeology, paleontology, botany and zoology.**

Did the permission you obtained from a local authority to perform the study include an agreement on access to outputs and benefit sharing? This may include procedures to enable fair distribution of the benefits and resources arising from the research performed. Please include any details of Prior Informed Consent and Benefit Sharing Agreements obtained. These may be required by field-specific regulations, for example the Convention on Biological Diversity (CBD) and the associated Nagoya Protocol.

No formal agreements on access to outputs or benefit sharing were established. However, the commercial supplier that provided the postmortem chicken breast samples receives access to the technical information generated from the study, including physicochemical and dielectric characterization data, as well as any subsequent technological insights that may arise from the research. Since the study involved only commercially obtained animal tissues and did not fall under regulations such as the Convention on Biological Diversity or the Nagoya Protocol, no additional consent or benefit-sharing mechanisms were required.

If the material used in your study was imported, please A) provide the year it was imported and B) indicate whether permits were obtained to import/export the materials used, C) provide details of any permits obtained. If this information is not available, please indicate this.

The biological samples used in this study were obtained locally from Multiservice Company DIR EIRL (Chota, Cajamarca, Peru) during the second quarter of 2024. Therefore, no import or export procedures were involved, and no permits were required.

If you used archival specimens, please state how the material used in your study was acquired by the institute it is held in and provide details of any permits obtained for the original excavations/ sample collection. If this information is not available, please indicate this.

No archival specimens were used in this study. All samples were obtained directly from the commercial supplier, and no historical collections or previously archived materials were involved. Therefore, no permits related to prior excavations or sample collection were applicable.

How was the potential cultural significance of the materials collected in your study to local communities considered in your research design? Were Indigenous peoples and/or local researchers and institutions involved with archaeological excavations / collection of specimens? If so, please provide a description of their involvement.

The materials used in this study have no cultural significance to local communities, as they consisted exclusively of commercially supplied chicken breast samples. No Indigenous peoples, local communities, or archaeological teams were involved in the collection of specimens. Since the study relied solely on postmortem animal tissues obtained through standard commercial processes, cultural considerations were not applicable to the research design.

If your manuscript includes photographs of human remains please indicate whether authors obtained permission from descendants or affiliated cultural communities to do so.

N/A
